# Linking Tissue Damage to Hyperspectral Reflectance for Non-Invasive Monitoring of Apple Fruit in Orchards

**DOI:** 10.3390/plants10020310

**Published:** 2021-02-05

**Authors:** Alexei Solovchenko, Alexei Dorokhov, Boris Shurygin, Alexandr Nikolenko, Vitaly Velichko, Igor Smirnov, Dmitriy Khort, Aleksandr Aksenov, Andrey Kuzin

**Affiliations:** 1 Michurin Federal Scientific Center, 393766 Michurinsk, Russia; shu_b@mail.ru (B.S.); andrey.kuzin1967@yandex.ru (A.K.); 2 Faculty of Biology, Lomonosov Moscow State University, 119234 Moscow, Russia; 3 Federal Scientific Agroengineering Center VIM, 109428 Moscow, Russia; dorokhov.vim@yandex.ru (A.D.); rashn-smirnov@yandex.ru (I.S.); Dmitriyhort@mail.ru (D.K.); 1053vim@mail.ru (A.A.); 4 Moscow Institute of Physics and Technology (National University), Phystech School of Aerospace Technologies, 117303 Moscow, Russia; alex_nikolenko@mail.ru; 5 Stavropol Fruit Nursery Center Plodoobjedinenie “Sady Stavropolya”, 357255 Stavropol, Russia; vit-velichko@mail.ru

**Keywords:** hyperspectral imaging, sunscald, scab, vegetation indices, pigments

## Abstract

Reflected light carries ample information about the biochemical composition, tissue architecture, and physiological condition of plants. Recent technical progress has paved the way for affordable imaging hyperspectrometers (IH) providing spatially resolved spectral information on plants on different levels, from individual plant organs to communities. The extraction of sensible information from hyperspectral images is difficult due to inherent complexity of plant tissue and canopy optics, especially when recorded under ambient sunlight. We report on the changes in hyperspectral reflectance accompanying the accumulation of anthocyanins in healthy apple (cultivars Ligol, Gala, Golden Delicious) fruits as well as in fruits affected by pigment breakdown during sunscald development and phytopathogen attacks. The measurements made outdoors with a snapshot IH were compared with traditional “point-type” reflectance measured with a spectrophotometer under controlled illumination conditions. The spectra captured by the IH were suitable for processing using the approaches previously developed for “point-type” apple fruit and leaf reflectance spectra. The validity of this approach was tested by constructing a novel index mBRI (modified browning reflectance index) for detection of tissue damages on the background of the anthocyanin absorption. The index was suggested in the form of mBRI = (*R*_640_^−1^ + *R*_800_^−1^) − *R*_678_^−1^. Difficulties of the interpretation of fruit hyperspectral reflectance images recorded in situ are discussed with possible implications for plant physiology and precision horticulture practices.

## 1. Introduction

Light reflected by plants carries ample information about its biochemical composition, tissue architecture, and physiological condition. Developmental changes in pigment composition as well as those induced by the environmental stresses and attacks of phytopathogens, manifest themselves as specific changes in plant reflection properties [1,2,3,4,5]. Thus, chloroplasts of mature apple peel cells possess photosynthetic pigments including chlorophylls *a*, *b* and carotenoids [4,6], so these cells are capable of photosynthesizing at a rate close to that documented in leaves [7]. Carotenoids are important for light harvesting and for photoprotection [8]. Anthocyanins responsible for the red color of apples increase their consumer acceptance [9] serving also as a protection against excessive solar radiation [10,11]. Non-destructive assessment of pigments in plants in general as well as in apple fruits is complicated by overlapping absorption spectra of individual pigments and the non-linear relationship of reflectance vs. pigment content [2,12].

In spite of the difficulties mentioned above, diverse approaches have been developed to obtain insights into plant leaf [2,3,4,5,13,14] and fruit [15] structure and function from multi- and hyperspectral reflectance data. It became clear that non-destructive assessment of plant phenotypic traits requires a comprehensive understanding of plant tissue optical features and spectroscopy of pigments in situ. A general approach to the analysis of reflectance spectra of plants implies the investigation of the variation of reflectance arising in response to changes in biochemical composition and morphology of plant tissues. This analysis aims at finding the spectral bands with the maximum sensitivity to the plant trait in question such as pigment content and/or the development of disorders [16,17,18,19,20]. However, recent studies rely mostly on the machine learning algorithms and the observed spectral features [21] and frequently ignore the underlying biological processes.

Although the overwhelming majority of plant tissue optics studies have been carried out on leaves [22], in the present work we employed apple fruit as a model. Apples exhibit highly resolved reflectance spectra due to the localization of the bulk of their pigments in a thin outer layer (so called “peel”) on highly reflective tissues with a low pigment content (the “pulp”) [12,23]. Another reason for using an apple as a model is its vast importance as a major fruit crop. In practice, it takes a lot of time and manual labor to sort out fruits with symptoms of damages and disorders with no market value and unsuited for storage. Attempts to apply optical reflectance spectroscopy for assessment of fruit quality and their physiological state have been undertaken for decades, but the key achievements belong mostly to the field of postharvest processes, e.g., grading, sorting, storing of fruits (see [1,15] and references therein). The progress in the pre-harvest non-invasive assessment is still modest although remarkable exceptions exist [5].

Visible-Near Infrared (Vis-NIR) spectroscopy is now a widespread tool that has emerged from the field of remote sensing. Current understanding of plant spectral features stems mostly from the “point-type” measurements with conventional spectroradiometers and spectrophotometers [15]. Recent technical progress has paved the way for affordable imaging hyperspectrometers (IH) providing spatially resolved spectral information on plants at different scales, from plant organs (leaves and fruits) to individual plants and ecosystems [5,24]. In the past two decades, the HRI technology has evolved into a powerful noninvasive inspection tool. However, the overwhelming majority of the studies on application of Vis-NIR spectroscopy is focused on postharvest applications where the basics of the spectroscopic and chemometric theory are established and a broad range of commercially available instrumentation for packing lines is available [25]. The studies dedicated to the spectral imaging of fruits in the field and interpretation of reflectance images of fruits recorded under ambient conditions are much scarcer. This is not surprising since the extraction of sensible information from hyperspectral reflectance images (HRI) might be difficult due to the inherent complexity of plant tissue and canopy-level optics [1]. These problems are exacerbated when the reflectance images are acquired outdoors under ambient sunlight. There were also doubts that IH can be helpful for monitoring eco-physiological responses even under controlled conditions [26]. These uncertainties and limitations stem particularly from an insufficient understanding of the relationships between biochemistry and architecture features of plant tissues and their measured optical properties [25]. Solving these problems is a key to achieve confident interpretation of reflectance images, development of computer vision and robotic systems for automated fruit harvesting and grading capable of working in real orchards [5,15].

Here, we report on the changes in reflectance spectra accompanying stress acclimation of and damages to apple fruits documented with an IH. Special attention was paid to the changes of the reflectance patterns and features accompanying transformation of pigments during accumulation of anthocyanins, pigment breakdown during photooxidative damage (sunscald) and cell necrosis after phytopathogen attacks. Striving for greater confidence of interpretation of the HRI recorded in situ, we compared features of reflectance comprising HRI with those from reflectance spectra measured with a conventional spectrophotometer under laboratory conditions, wet biochemical analyses, and microscopic observations. 

## 2. Results

**Figure 1:**
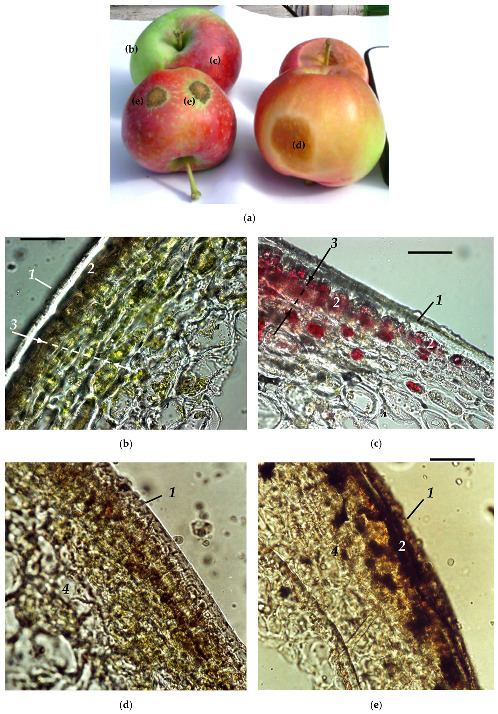
Visual appearance (**a**), anatomy and morphology of healthy (**b**,**c**) green (**b**) and red (**c**) apple fruit as well as fruit affected by (**d**) mild sunscald and (**e**) severe cell necrosis due to scab. In panel (a), the fruit surface regions corresponding to the micrographs (b–e) are shown. *1*—cuticle, *2*—epiderm, *3*—pigment-enriched compact “peel” (outer mesocarp), *4*—highly reflective “pulp” (inner mesocarp). The images were taken using Zeiss Axioscope photomicroscope at a magnification of ×200. Scale bar: 50 µm.

For this study, fruits bearing no visual symptoms of damage were selected as well as fruits affected to a different extent by physiological disorders commonly encountered in orchards (sunburn and sunscald) and phytopathogen lesions (lesions, cell necroses, and cracks from apple scab, *Venturia inaequalis*) (Figure 1a). The fruits showing chlorophyll loss (discoloration) and slight browning symptoms were designated as “mildly affected”. The fruits with dark-brown patches and pronounced tissue necroses were designated as “severely affected” either by sunscald or apple scab. We obtained the hyperspectral reflectance features of healthy and damaged apple fruit traceable to the characteristic features of the fruit visual appearance and morphology. Towards this end, the same fruits were used for recording the HRI under ambient conditions and for conventional point-type spectral reflectance measurements (see Section 5 below).

### 2.1. Visual Appearance and Microscopic Observation of Healthy Apple Fruit Tissue and Damages

The studied fruits of apple, *Malus* × *domestica* Borkh displayed typical anatomical features of apple fruit (Figure 1b–e). The fruit is covered by cuticle, the first structure of plant surface which interacts with environmental factors including solar radiation [27,28]. Below the cuticle and a single layer of epidermal cells, there were several layers of relatively small cells harboring the bulk of chloroplasts and hence fruit pigments—chlorophylls (Chl) and carotenoids (Car) as well as anthocyanins (AnC) and colorless UV-absorbing phenolics in the cell vacuoles. Most of the fleshy part of the apple fruit constitutes the parenchymatous tissue, composed of large cells. It has a low pigment content but incorporates numerous spaces between its cells and numerous interfaces, and therefore this tissue is highly reflective [23]. 

Physiological diseases of a diverse nature are exemplified by sunscald [29] as well as by attacks of phytopathogens, e.g., apple scab (*Venturia inaequalis*) induces breakdown of pigments apparent as gradual discoloration in the apple tissues (Figure 1e; Table 1), a considerable part of the fruit crop is lost annually as a result [30]. The extent of sunscald development is strongly dependent on cultivar, climatic conditions, and agricultural practices [31]. The disruption of cell compartments triggers the oxidation of the vacuolar phenols by polyphenol oxidase resulting in the formation of polymeric melanin-like pigment(s) production [32] manifested by progressive browning of the fruit tissues. On the fruit tissues section, browning was evident as cell necroses and compact dark inclusions filling the protoplast of the affected cells (Figure 1c,d).

### 2.2. Characteristic Features of Healthy Apple Fruit Reflectance

**Figure 2:**
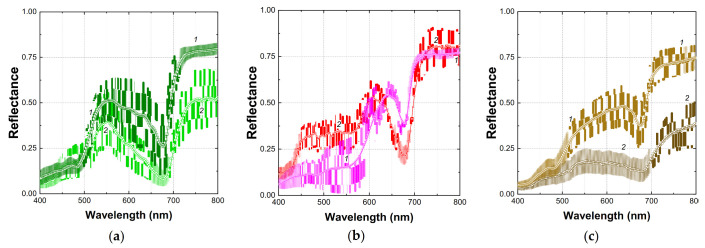
Typical reflectance spectra of (**a**) healthy green; (**b**) healthy red, and (**c**) damaged brown apple fruit surface regions. Spectra measured by conventional spectrophotometer (Agilent Cary Bio 300) are shown (curves *1*) together with those extracted from hyperspectral reflectance images recorded with SPECIM IQ snapshot hyperspectral camera at ambient illumination (curves *2*) together with their standard deviation (shaded areas). Corresponding raw reflectance spectra can be found in the supplementary data file.

Visible-NIR (Vis-NIR) reflectance spectra of apple fruits taken with a conventional spectrophotometer and those extracted from the HRI are shown in Figure 2. A common feature of apple fruit is a high reflectance in the near infra-red (NIR) region of the spectrum (75–85% in the range 750–800 nm). A characteristic feature of the fruit bearing no visual symptoms of damages was an almost flat reflectance spectrum in the NIR (720–850 nm). Notably, the increased variation in the NIR region of the reflectance spectra from the HRI was detected (Figure 2a, curve *2*) whose values occasionally exceeded the unity in this range.

In the visible range, the spectral features attributable to the absorption by Chl, Car, and (in the red-colored fruit) AnC were evident in the spectra of apple fruits (for detailed description of the reflectance spectral features of apples, see [12,23]). In the red part of the visible region, broad bands of Chl *a* (the pronounced minimum near 678 nm) and Chl *b* absorption (a shoulder near 650 nm) were evident in fruits regardless of their color. In the blue-green part of the visible region of the spectrum, apple fruit reflectance was low due to the combined absorption of Chl, Car, and, in red-colored fruit, AnC (Figure 2a,b). Accumulation of AnC manifested itself as a characteristic shoulder near 550 nm in the case of AnC-containing red-colored fruits (Figure 2b). An increase in the intensity of the red coloration (and corresponding AnC content) was accompanied by a decline of reflectance in the range 600–400 nm, resulting in a shift of the green edge position toward longer wavelengths and a progressive broadening of the reflectance minimum attributable to AnC. In the short-wave part of the Vis range, a decline in reflectance due to the tailing contribution of phenolic compounds possessing the main absorbance peaks in UV [23] was occasionally observed. The reflectance features of healthy apple fruit mentioned above were in line with the result of analytical pigment assay (Table 1). The red-colored fruits featured a high AnC content whereas in the yellow-green fruits AnC were hardly detectable. At the same time, the healthy red fruits featured a lower content of Chl and Car but a higher flavonol content as compared with the healthy green apples.

**Figure 3:**
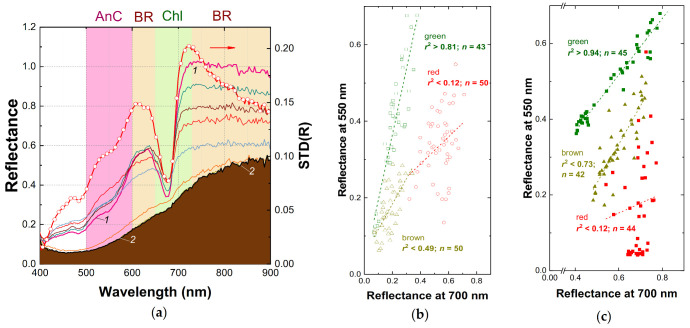
Hyperspectral reflectance spectra (**a**) taken with the SPECIM IQ imaging hyperspectrometer from the regions of a representative apple fruit (cv. Ligol) featuring a different degree of sunscald-induced browning ranging from visually unaffected (curve *1*) to severely affected (curve *2*) (left scale) and their standard deviation, STD (symbols, right scale). Panels (**b**,**c**): relationships between reflectance coefficients in the green, *R*_550_ and in the red edge, *R*_700_ spectral regions in healthy green fruits (squares), red fruits (circles), and fruits affected by browning (triangles) plotted for the hyperspectral data (**b**) extracted from the HRI images and (**c**) for the “point-type” reflectance measurements. In panel (**a**), the spectral regions governed by chlorophyll (Chl), anthocyanin (AnC), and melanin-like brown pigment absorption (BR) are shown [12,30].

Notably, a fundamental feature of plant reflectance previously described for numerous plant species, the close correlation of reflectance values at 550 nm and 700 nm, was evident in the spectra of yellow-green fruits lacking AnC [12,33], (*r*^2^ > 0.99, Figure 3b) regardless of their Chl content. In the AnC-containing healthy red apple fruit reflectance at 550 nm, *R*_550_ was considerably lower compared to that at 700 nm, *R*_700_. As a result of AnC absorption, a strong correlation of *R*_550_ and *R*_700_ was lost in the red fruits (Figure 3b).

### 2.3. Reflectance Spectral Signature of Damaged Apple Fruit Tissues

In this work, the reflectance spectra of the apple fruits damaged by a physiological disorder (sunscald) and a phytopathogen attack (apple scab) have been acquired using a conventional spectrophotometer (Figure 2c). The spectra of the damages induced by the physiological disorder and the phytopathogen were similar (Figure S2): both displayed a decline of reflectance in the NIR and the green regions of the spectrum along with the bleaching of the main absorption band of Chl in the red. Therefore, these spectra have been considered as a single dataset in the following analysis. The “point-type” spectra of the damaged apple fruits were compared with those taken with an IH (Figure 2c). The spectra of damaged fruit obtained in this study displayed a spectral signature of the browning pigments previously documented in apples affected by sunburn and superficial scald [30]. The development of damage symptoms visually apparent as discoloration and brown patches on the fruit surface led to dramatic changes in their reflectance spectra (Figure 2c and Figure 3a). As damage progressed, reflectance of the affected fruits decreased in the whole spectral range studied (400–1000 nm) and the spectral features of Chl (in the band 620–700 nm) and Car (a sharp increase in reflectance near 500 nm) were flattened out regardless of AnC presence. A considerable decrease of reflectance occurred in the NIR, more pronounced at shorter-wavelength parts of the NIR region of the spectrum. However, the highest variation in reflectance recorded in presence of AnC occurred in the range 600–650 nm (Figure 3a). Overall, an increase of reflectance variation in the NIR and in the green-to-orange regions of the spectra are characteristic features of the disorders accompanied by browning [30,34]. Another important feature of changes in the reflectance spectra accompanying the development of browning was a low variation of reflectance coefficients in the band of long-wave Chl absorption maximum centered at 678 nm (Figure 3a). The region of the strong combined absorption of all pigment groups in the blue-green region of the spectrum was also characterized by a low variation of reflectance (400–500 nm; see the STD spectrum in Figure 3a).

The results of the biochemical piment assay were generally in agreement with the observed changes of the fruit reflectance during development of the damages (Table 1). Both types of damages (sunscald and apple scab) studied induced a sharp decline in photosynthetic pigment (Chl and Car) contents. The mild sunscald was characterized by the buildup of flavonols (but not AnC) in the affected fruit tissues. By contrast, the advanced stages of the damages we manifested by a decline in AnC and flavonol contents. 

### 2.4. Spectral Indices for the Processing of Hyperspectral Reflectance Images

To develop a sensitive optical indicator of plant damages accompanied by browning, a previously developed methodological framework for the construction of vegetation indices for the assessment of plant pigments was used [35]. According to this framework, it was necessary to select the spectral bands exhibiting (i) a high sensitivity to browning pigment and (ii) a minimal sensitivity to browning, but sensitive to contributions by Chl and Car. 

**Figure 4:**
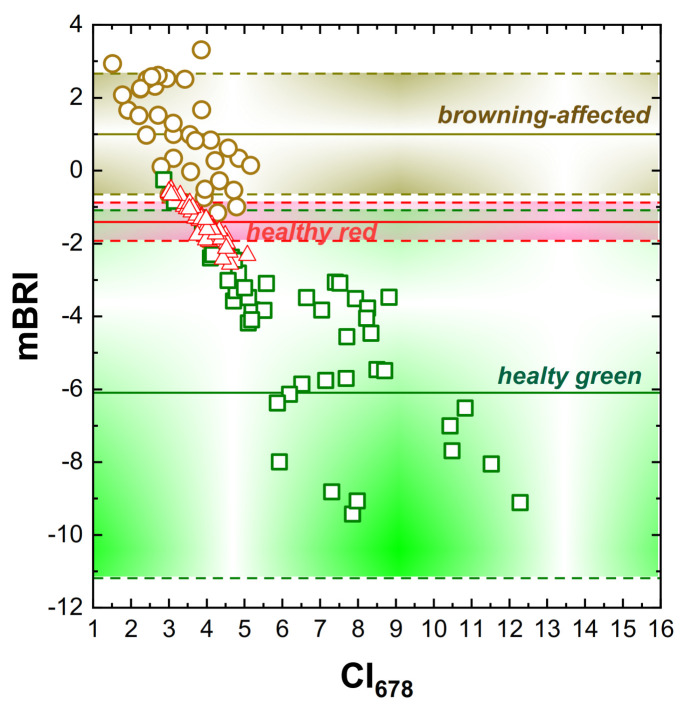
Comparison of the values of mBRI index calculated on the basis of hyperspectral reflectance images captured by the IH for healthy green (squares; –6.09 ± 5.00, *n* = 43), healthy red (triangles, –1.41 ± 0.52, *n* = 50), and those affected by scab- and scald-induced browning (circles; 1.00 ± 1.66, *n* = 50) apple fruits plotted vs. CI_678_—the ratio *R*_800_/*R*_678_, a proxy of Chl [12]. The average values ± STD are represented on the plot as solid and dashed horizontal lines, respectively. All the averages were significantly different according to the Student’s *t*-test at the level of *p* < 0.05. The corresponding raw spectra can be found in the supplementary data file; see also Figure S3.

As it follows from the analysis of the reflectance spectra presented above, *R*_NIR_ and *R*_640_ exhibited a high sensitivity to browning manifested as a large decline along with the development of the damage. In the NIR region of the spectrum, the channel of 800 nm was selected since it is far from the long-wave Chl absorption maximum so the interference from this pigment would be small [12]. At the same time, the contribution of the brown pigments at this wavelength is still high ([30], see also Figure S3a). Since reflectance of leaves and fruits exhibits inverse non-linear relationships to their pigment content inverse values, we suggested *R*_640_^−1^ and *R*_800_^−1^ as the terms sensitive to accumulation of the browning pigments and *R*_678_^−1^ as a term with small sensitivity to browning but sensitive to Chl content in apple (see e.g., [12]). Subtracting *R*_678_^−1^ as a proxy to Chl concentration from the sum (*R*_640_^−1^ + *R*_800_^−1^) allowed us to construct an index indicative of the development of browning (Figure 4). Reflectance at 800 nm, which exhibits a low variation in healthy fruits but decreases significantly in the course of browning (Figure 2 and Figure 3a) was introduced as a term, increasing its sensitivity to browning. Indeed, grayscale images in the near-infrared (800 nm) channel contained distinct images of the damaged spots (Figure S4a), but the information in this channel *per se* was insufficient for the robust differentiation of the damaged fruit regions (Figure S3a), likely due to a variable outdoor illumination condition. Finally, the modified browning reflectance index (mBRI) was suggested in the form:

mBRI = (*R*_640_^−1^ + *R*_800_^−1^) – *R*_678_^−1^, (1)

where *R*_640_, *R*_800_, and *R*_678_ are the reflectance coefficients in the bands indicated by the subscripts.

Generally, the healthy fruits exhibited mBRI values below −1.0 regardless of their Chl and AnC content, whereas fruits even slightly affected by browning possessed mBRI values above −0.9 (Figure 4). The average values of the mBRI index calculated for the affected fruits differed significantly (*p* < 0.05) from those for the healthy read and healthy green. 

**Figure 5:**
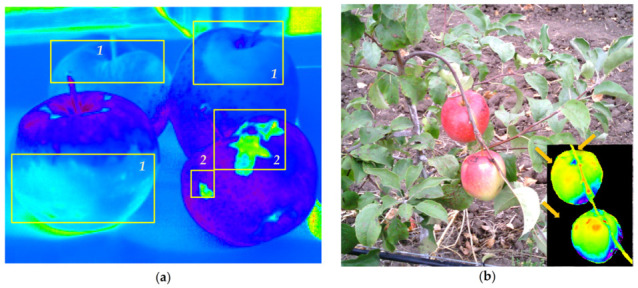
Using the mBRI index for the detection of damages in the HRI images of Ligol apple fruits obtained at ambient illumination. (**a**) Regions of apple fruit affected by (*1*) sunscald-induced browning and (*2*) scab-induced necroses highlighted in the false-color images based on the calculated values of the mBRI index. (**b**) RGB images reconstructed from a HRI obtained in a commercial orchard. Inset: the same scene, in masked false-color, reflecting the mBRI values revealing the onset of sunscald-induced damages (arrows). Violet-to-blue colors correspond to the low mBRI values (healthy tissues), green colors correspond to intermediate mBRI values (moderately affected tissues), and yellow-to-red colors correspond to high mBRI values (severely affected tissues).

**Figure 6:**
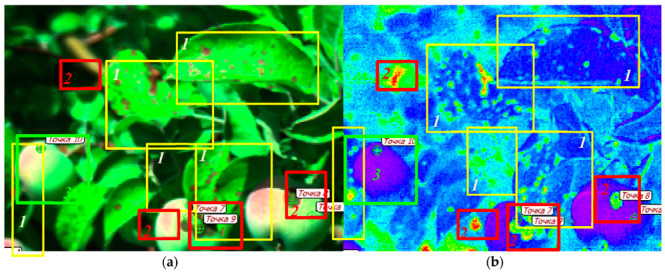
Using the mBRI index for the detection of profound apple scab-induced damages in the HRI images of unripe Ligol apple fruits and leaves obtained at ambient illumination (see also Figure S4). (**a**) The scene in RGB composite color (as perceived by the human eye and reconstructed from a HRI obtained in a commercial orchard). (**b**) The same scene in false color representing the calculated values of the mBRI index. The magenta-blue regions represent unaffected leaf and fruit area, the green-red areas are affected by the scab-induced browning. The rectangles outline the damaged leaves (*1*), fruits (*2*) as well as a healthy fruit (*3*) in a and b. Note the fruit damages (in the left) is hardly visible on the RGB images due to shading but readily discernible in the mBRI index image. Violet-to-blue colors correspond to the low mBRI values (healthy tissues), green-to-red colors correspond to high mBRI values (severely affected tissues).

Therefore, the index mBRI could be used for quantitative assessment of sunscald and other disorders accompanied by browning as well as plant diseases affecting the close relationships between reflectances at 550 and 700 nm using the HRI images acquired in this work. It should be noted that the original index BRI developed by browning damage assessment [30] was not applicable for red-colored apple fruits since the presence of AnC in their peel causes a significant decrease of *R*_550_ interfering with the browning pigment assessment. The original index BRI was characterized by a poor performance when applied to the same HRI (see e.g., Figures S3b and S4b). By contrast, the index mBRI developed in this study performed reasonably well even in the case of AnC-containing fruits (Figure 4, Figure 5 and Figure 6). 

## 3. Discussion

Reflected light signal conveys a plethora of valuable information on the integrity and physiological condition of plants including their pigment content and composition, photosynthetic activity etc. [4] In the case of fruits, the information about the fruit carried by the reflected light also translates into the perceived fruit quality (appearance, ripeness, presence of damage) [1,15,25]. This study highlighted the effects of sunscald, a widespread photooxidative damage [36,37,38], and apple scab on hyperspectral reflectance images of apple fruit in orchards as compared with traditional “point-type” reflectance measurements. 

An important obstacle for the extraction of sensible information from HRI is related to a high degree of fruit heterogeneity, also in terms of their optical properties, exacerbated by volatile illumination conditions during the outdoor data recording. The heterogeneity of the fruits arises as an interactive effect of diverse internal and external factors affecting the fruits during their development in orchards. However, our previous analysis of the “point-type” reflectance spectra revealed patterns indicative of the development of healthy fruit and damage to them [12,23,29,39,40]. We attempted to use these patterns to better understand the information content of the HRI obtained by an IH and leverage them for apple damage detection under ambient illumination conditions.

As a first step in this direction, we compared the reflectance spectra extracted from the HRI and the “point-type” spectra measured by the spectrophotometer equipped with an integrated sphere. The results confirmed the general similarity of the reflectance spectra from either source (Figure 2). This conclusion enabled us to employ the relationships previously documented for the point-type measurements of apple fruit reflectance to interpret the features of the HRI obtained in the field. The large variation of the infra-red region of the spectra taken with the IH stemmed from the variability of the incident flux of solar radiation which is unavoidable in the measurements under ambient conditions. To a certain extent, this variation was smoothed by normalization to the signal reflected by the reflectivity standard, but this correction was insufficient when the illumination changes were rapid as compared with the time of HRI recording (ca. 1 min under our experimental conditions). Nevertheless, as discussed below, the HRI were a suitable source of spectral data for detection of the apple fruit damages under ambient conditions.

The damage-induced features of the fruit reflectance extracted from the HRI were in agreement with those documented with “point-type” reflectance measurements. Generally, the changes in the apple fruit reflectance resembled the picture of spectral changes during Chl and Car photobleaching by strong light irradiation in leaves and fruits [29,40,41]. In sunscald-affected fruits, a simultaneous photodegradation of Car and Chl took place resulting in disappearance of the absorption bands of these pigments followed by the formation of melanin-like pigments manifesting themselves as a browning of the fruit surface. Paradoxically, the fruit tissues severely affected by browning possessed a relatively low flavonol content. Likely, this was due to the conversion of the bulk of phenolic compounds into the melanin-like browning pigments (see e.g., Figure 1e). We were unable to differentiate the spectra of fruit surface damaged by environmental stresses or the phytopathogen due to similarity of their fine structure and amplitude. This similarity can stem from similar chemistry of the brown pigment formation during the development of these damages [42]. There was also a decline in the reflectance in the blue-violet region of the spectrum related to the buildup of peel flavonols (mainly, rutin) in response to elevated UV fluxes coming with elevated fluxes of incident solar radiation [43,44].

At the next stage, we attempted to construct a spectral index for detection of browning in the HRI in real orchards. Towards this end, we employed a previously developed methodology of the construction of vegetation indices for leaf and fruit pigment assessment using reciprocal reflectance coefficients [12,17,45]. Briefly, to assess pigments absorbed in the green region of the spectrum such as AnC or the browning pigments, compensation of the interference from Chl is necessary. This can be done using reflectance in the red (678 nm) region of the spectrum, which is a proxy of Chl content [12]. Another problem, the interference from the overlapping absorption of AnC and brown pigments, was solved by shifting the spectral band of for the index from the green (550 nm) to the yellow-orange (640 nm) region where absorption of AnC and Chl is low, but the absorption of the browning pigments is significant. Adding an NIR band responsive to browning pigment accumulation (800 nm) increased the sensitivity of the index. The resulting index (Equation (1)) allowed us to distinguish the regions of fruit surface affected by sunscald and scab-induced necroses in HRI (Figure 4, Figure 5 and Figure 6). 

An interesting observation was comprised by a high (*r*^2^ > 0.79) inverse correlation of mBRI and a chlorophyll index based on the 678-nm channel (CI_678_), the *R*_800_/*R*_678_ ratio representing a proxy to apple peel Chl content [12]. Obviously, this correlation cannot be a direct manifestation of Chl variation since the mBRI is corrected for the contribution of the absorption of light by Chl. It is more likely that this relationship is an indirect consequence of a stronger decline in the photosynthetic pigment content in fruits with a stronger damage also presuming a higher content of the brown pigments in these fruits.

One can argue that in many cases the damages can be seen on “simple” RGB images (see, e.g., Figure 6a) so the use of an IH would be redundant. However, leveraging the RGB images needs advanced approaches to (i) detect and count the damages and (ii) to eliminate interferences from non-relevant objects (like sky, soil, tree trunks) inevitably getting onto the photographs. This is possible with modern machine learning algorithms, e.g., artificial neural networks, ANN (a good example is described in [5]). Still, this approach requires a labor-intensive training of the ANN to take down the error rate of the neural network-based algorithm to the minimum. Furthermore, one can easily spot advanced stages of the damages but not the initial stages of damages, especially those of sunscald. Weakly expressed sunscald (called at this stage “sunburn”) is easily overlooked but it is important to sort out affected fruits before loading the batch to a storage chamber, since the affected fruit emit ethylene is at an increased rate as compared with healthy fruits [37,39,46]. This process promotes fruit ripening facilitating storage disorders, secondary infections, and shortening the fruit shelf life [47]. Obviously, those damages can be detected more reliably with the using of spectral hints like the mBRI index developed in this study. Thus, a small but measurable increase in mBRI value took place even when the affected fruit surface revealed little or no visually detectable symptoms of the injury. In our opinion, “hybrid” methods (those combining the advantages of spectral detection and machine learning) are among the most promising for the detection of damages. An established spectral detection method would not likely require the full spectral resolution of IH. However, having the full set of hyperspectral data is essential for finding the suitable spectral bands during the development of such a method. Thus, only three spectral channels (NIR, red, orange) were sufficient to achieve the goal in this study, but these spectral channels were not known *a priori* and have to be selected from the full-resolution hyperspectral dataset.

## 4. Conclusions

Collectively, the obtained findings allowed us to bridge the gap between point-type and field imaging-based measurement approaches, at least for the detection of damages associated with tissue browning. The comparative analysis has been focused on distinguishing the spectra of healthy fruits from those affected by physiological and phytopathogen-induced damages. The results support the compatibility of the HRI data obtained in the orchard under ambient illumination conditions using a portable IH with the fruit reflectance measured under controlled conditions with bench-top double-beam spectrophotometers. Both approaches provided sufficient detail on reflectance patterns characteristic of AnC accumulation, photooxidative injury (sunscald), and cell necrosis after phytopathogen attacks. Particularly, the fundamental correlation of reflectances at 550 nm and 700 nm (and loss thereof in the red and/or damaged fruits) was discovered in the reflectance spectra extracted from HRI. Despite the difficulties of the reflected signal normalization under outdoor conditions, the spectral data originating from the HRI captured in the field can be processed within the framework earlier developed for the analysis of “point-type” apple fruit spectra and, more generally, vegetation reflectance spectra [33,43]. The validity of this approach is supported by the possibility to construct a modified browning reflectance index, mBRI. This index provided for a detection of damages manifesting themselves as tissue browning on the hyperspectral images even in AnC-containing red fruits. Our findings facilitate extending the HRI-based proximal monitoring beyond the postharvest technology domain further to industrial apple orchards complementing the modern machine learning-based approaches, although more research is required. On-tree ripeness assessment taking into account the inherent heterogeneity of fruit physiological conditions constitutes an example of hot problems that can be potentially solved using the HRI methodology.

## 5. Materials and Methods 

### 5.1. Plant Material and Measurement Workflow

Fruits of apple (*Malus* × *domestica* Borkh.) cultivars Golden Delicious (green-colored), Ligol and Gala (red-colored) with or without symptoms of damage by sunscald or apple scab were visually selected (for the detailed information on samples, see Table 2). Fruits were grown either at an experimental orchard of Michurin Federal Scientific Center (Michurinsk, Tambov region, Russia) or in the production orchard of the “Sady Karachaevo-Cherkesii” fruit growing company (Karachay-Cherkess Republic, Russia).

The fruits were first imaged with the IH while they were attached to the tree. Afterwards the fruits were hand-picked and the side of fruit facing the IH during the imaging procedure was marked. In certain cases, additional HRI images were acquired under ambient illumination. Then the fruits were transferred to the laboratory within one hour after picking where reflectance of the same side that was imaged with the IH was measured with a conventional spectrophotometer (“point”-type measurements). Finally, the light microscopy study has been carried out followed by the pigment assay using the same apple surface areas that were measured in the previous step. The microscopic investigation and the biochemical assays were completed within two to three hours from the fruit picking.

### 5.2. Spectral Reflectance Measurements

#### 5.2.1. Hyperspectral Reflectance Imaging

The hyperspectral reflectance data-containing images were captured with a snapshot imaging hyperspectrometer IQ (SPECIM, Oulu, Finland). The measurements were conducted from 10:00 a.m.–11:00 a.m. For each pixel of the hyperspectral image (512 × 512 pixels), a reflectance spectrum (spectral range 400–1000 nm; spectral resolution 1 nm) was recorded against a reflectivity standard made of Spectralon^®^ under ambient illumination. The average intensity of solar radiation during the measurement period was 850 µmol PAR quanta m^–2^ s^–1^ as sensed by a LI-850 quantum meter (LiCOR, Lincoln, NE, USA). For the construction of the spectral index, an “investigation” dataset has been prepared from the individual spectra. These spectra were manually selected and sampled from the HRI images of visually homogenous fruit regions bearing signs of the damages or lacking them (designated as “healthy green”, “healthy red”, and “damaged”, see Figure 1a). Overall, 143 spectra have been sampled for the “investigation” dataset (see the supplementary data file). The sampling of the spectral data and rendering of the index images have been carried out using Gelion, the original software for processing hyperspectral images (https://github.com/AlexanderMipt/Gelion).

#### 5.2.2. Reflectance Spectra Measurement with a Conventional Spectrophotometer

Diffuse reflectance spectra of the apple fruits were recorded at 400–800 nm range with an Agilent Cary Bio 300 (Agilent, Santa Clara, CA, USA) spectrophotometer equipped with an integrating sphere attachment (internal diameter 100 mm) against a Spectralon^®^ plate as a 100% reflectivity standard. The diameter of the input port of the integrating sphere was 12 mm corresponding to the measured area of ca. 3.8 cm^2^. The measured spectra can be found in the supplementary data file. Six fruits damaged during picking and transportation to the laboratory have been excluded from this phase of analysis, so the “point-type” spectra dataset contained 137 spectra (Table 2).

### 5.3. Light Microscopy

The light microphotographs of hand-made cross-sections of fruit peel samples were taken with an Axioscope (Karl Zeiss, Jena, Germany) microscope fitted with an MRC digital camera (Carl Zeiss, Jena, Germany).

### 5.4. Pigment Assay

The assay of total chlorophylls, carotenoids, anthocyanins, and flavonols in an extract from apple sample zone used for reflectance measurements was carried out essentially as described in [48]. Briefly, fruit peel disks (total area of 3.8 cm^2^, thickness ca. 1 mm) made with a cork borer and a scalpel were ground in chloroform–methanol (2:1, *v*/*v*) in the presence of 100 mg MgO. After completion of extraction, homogenates were filtered through a pre-soaked with the chloroform–methanol mixture paper filter, and distilled water (1/5 of total extract volume) was added. Then extracts were centrifuged at 3000× *g* with a 5804R (Eppendorf, Hamburg, Germany) centrifuge for 10 min until phase separation. Total chlorophyll and carotenoid concentrations were quantified spectrophotometrically with an Agilent Cary Bio 300 spectrophotometer in the lower (chloroform) phase using coefficients reported by Wellburn [49]. The upper (water–methanol) phase was used for assay of total flavonols, which were quantified spectrophotometrically using the band around 358 nm where flavonols exert the dominant contribution to the absorption and molar absorption coefficient ε_358_ = 25.4 mM^−1^ cm^−1^ determined for rutin in 80% aqueous methanol. After determination of flavonols the water–methanol phase was acidified with HCl (final concentration of HCl = 0.1%) and used for quantification of anthocyanins. Anthocyanins were assayed by measuring absorbance at 530 nm; an absorption coefficient of 30 mM^−1^ cm^−1^ [50] was accepted for these pigments. Pigment contents were expressed on a fruit surface basis.

### 5.5. Statistical Treatment

Average values are shown in the figures and tables with the corresponding standard deviation values if not indicated otherwise. A significance of difference between the averages, as indicated in the figures and tables, was estimated using Student’s *t*-test in Origin 2019 (OriginLab, Northampton, MA, USA) or ANOVA using Excel spreadsheet software (Microsoft, Redmond, WA, USA). The significance level of 0.05 has been accepted in the statistical treatment of the data.

## Figures and Tables

**Table 1:** Average pigment content of the apple fruits studied in this work.

Pigment Group	Fruit Condition ^1,2^				
	Healthy Green	Healthy Red	Sunscald (Mild)	Sunscald (Severe)	Scab-Affected
Chlorophylls	15.3 ± 1.65 ^a^	8.64 ± 2.14 ^a,b^	5.62 ± 2.23 ^b^	1.59 ± 0.83 ^c^	0.54 ± 0.31 ^c^
Carotenoids	4.72 ± 0.39 ^d^	2.92 ± 0.75 ^d.e^	4.74 ± 1.82 ^d,e^	2.59 ± 2.14 ^d,e^	1.32 ± 0.83 ^e^
Anthocyanins	0.35 ± 0.13 ^f^	36.1 ± 7.54 ^g^	5.21 ± 3.91 ^h^	5.3 ± 0.4 ^h^	2.75 ± 1.34 ^f,h^
Flavonols	29.1 ± 6.23 ^i^	151 ± 14.2 ^j^	185 ± 29.1 ^j^	25.6 ± 4.8 ^i^	15.9 ± 4.55 ^i^

^1^ For the information on the number of samples, see Table 2. ^2^ Averages ± standard deviations are shown. Different letters denote values significantly different at the level of *p* < 0.05, according to ANOVA.

**Table 2:** The number of apple fruits studied in this work.

Cultivar	Fruit Condition					
	Healthy Green	Healthy Red	Sunscald (Mild)	Sunscald (Severe)	Scab-Affected	Total
Ligol	0	39	6	10	14	69
Golden Delicious	45	0	4	5	0	54
Gala	0	5	2	7	0	14
Total	45	44	12	22	14	137

## Supplementary Materials

The following are available online at https://www.mdpi.com/2223-7747/10/2/310/s1, Figure S1: Typical RGB photos of the studied (a) red-colored “Gala” and (b) green-colored “Golden Delicious” healthy fruits on the trees made with a viewfinder camera of the SPECIM IQ snapshot imaging hyperspectrometer, Figure S2: Average reflectance spectra extracted from the HRI of the fruits affected by sunscald and apple scab with corresponding STD values, Figure S3: Average values of *R*_800_ and the indexes BRI and NDVI with corresponding STD values calculated for the scene shown in Figure 6 in the main text, Figure S4: alternative representations of the scene shown on Figure 6 in the main text in a NIR reflectance channel, *R*_800_; the index BRI, and (c) the index NDVI.
