# Supplementary material for: Linking Tissue Damage to Hyperspectral Reflectance for Non-Invasive Monitoring of Apple Fruit in Orchards
Source: Plants (Basel). 2021 Feb 5;10(2):310. doi: 10.3390/plants10020310 (PMC7914439; doi:10.3390/plants10020310)
Supplement: Supplementary file 1 [file plants-10-00310-s001.zip › plants-1059960-supplementary_revised.docx]

Supplementary figures

| 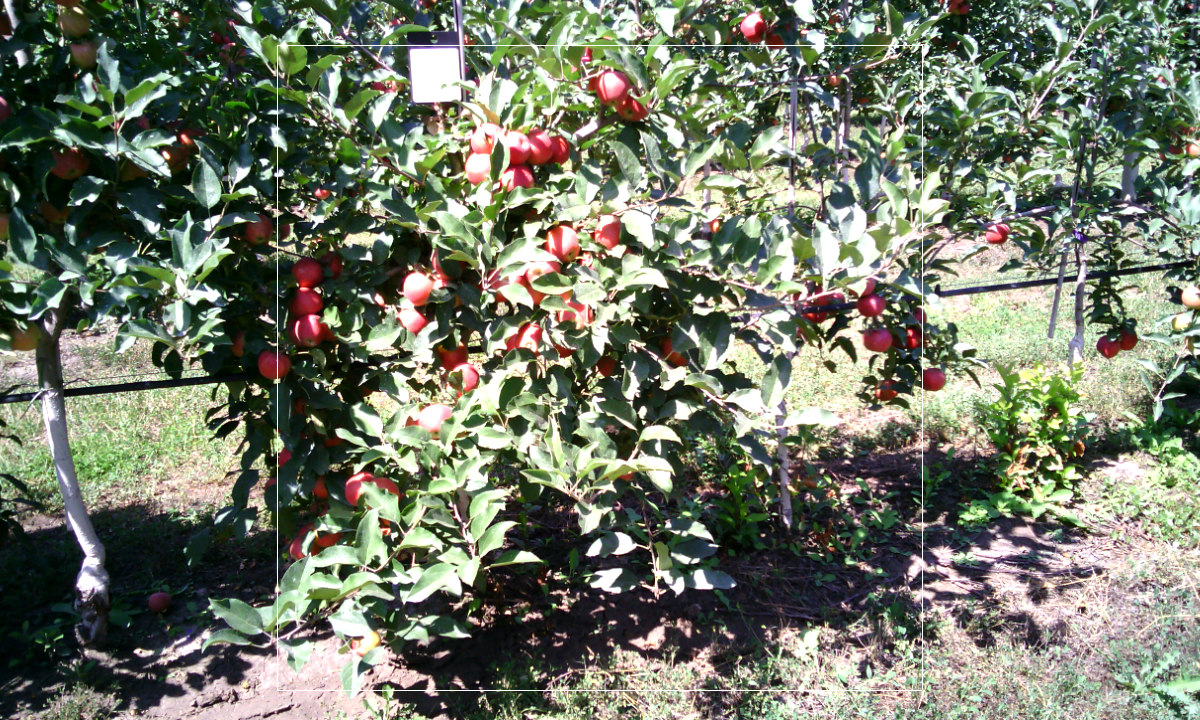  (**a**) | 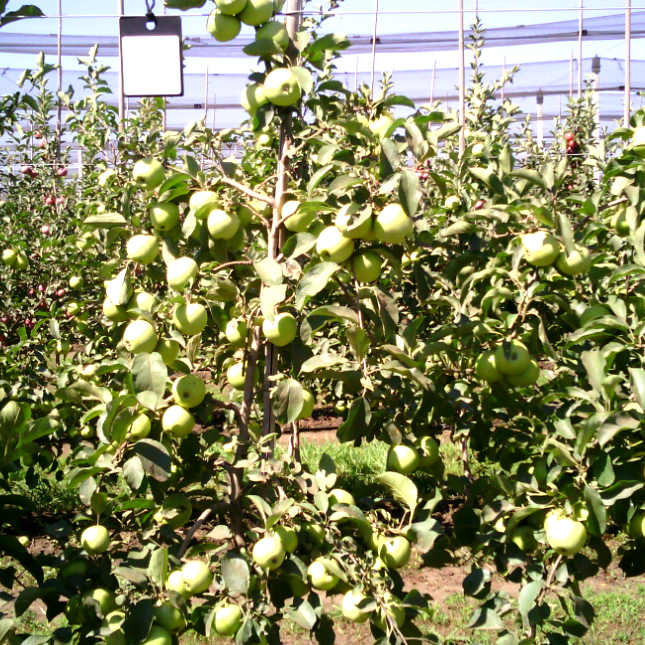  (**b**) |
| --- | --- |

**Figure S1.** Typical RGB photos of the studied (**a**) red-colored “Gala” and (**b**) green-colored “Golden Delicious” healthy fruits on the trees made with a viewfinder camera of the SPECIM IQ snapshot imaging hyperspectrometer (see Methods). Note the 100% reflectivity standard above the trees.

**Figure S2.** Average reflectance spectra extracted from the HRI of the fruits affected by sunscald (squares) and apple scab (circles) with corresponding STD values (shaded areas). See also Fig. 2c and 3 in the main text.

|   (**a**) |
| --- |
|   (**b**) |
|   (**c**) |

**Figure S3.** The averages ± STD for (**a**) *R*_800_, (**b**) BRI, and (**c**) NDVI calculated for the spectra extracted from different HRIs and used for calculation of the mBRI shown in Fig. 4 in the main text.

| 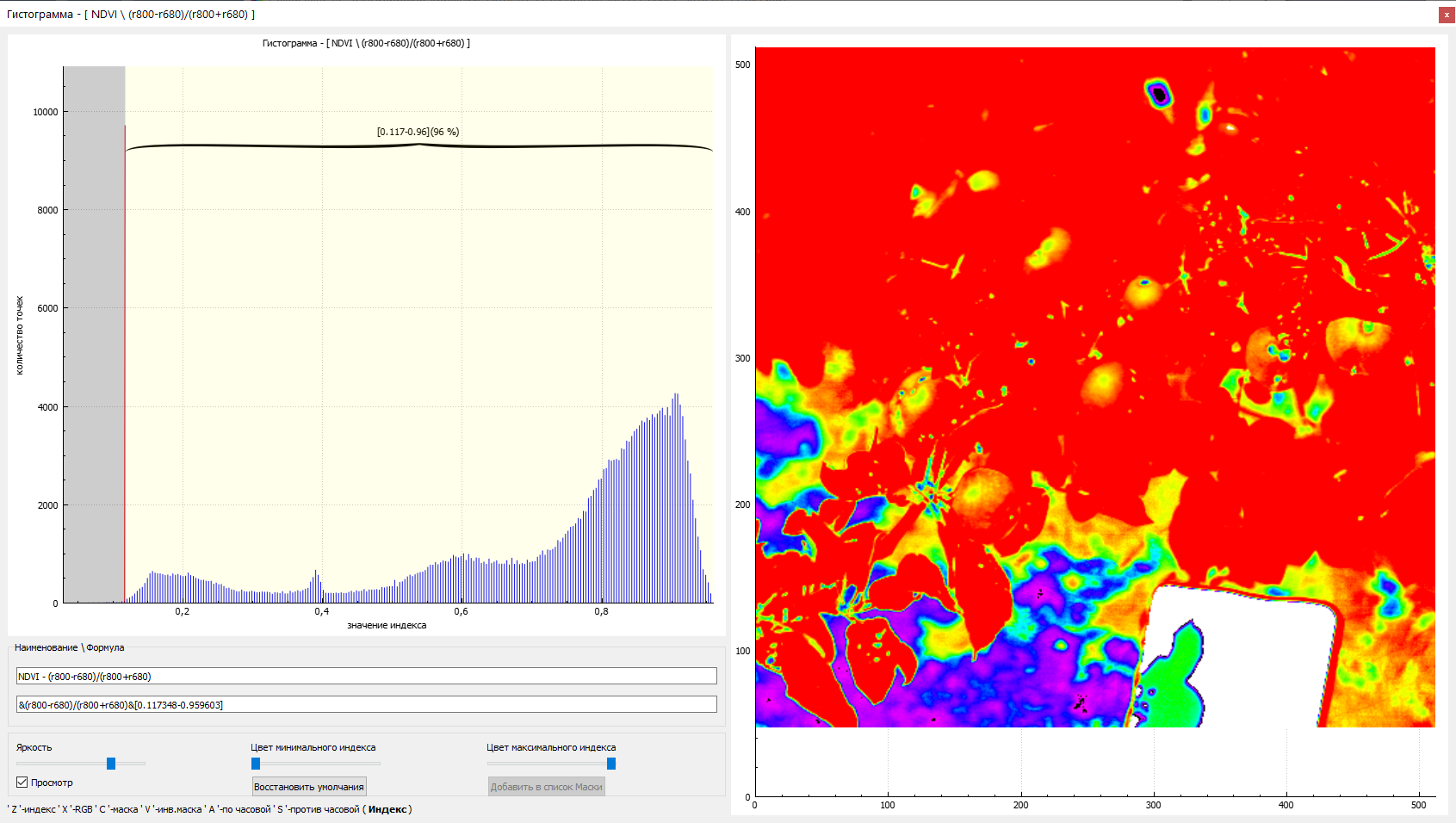  (**a**) |
| --- |
| 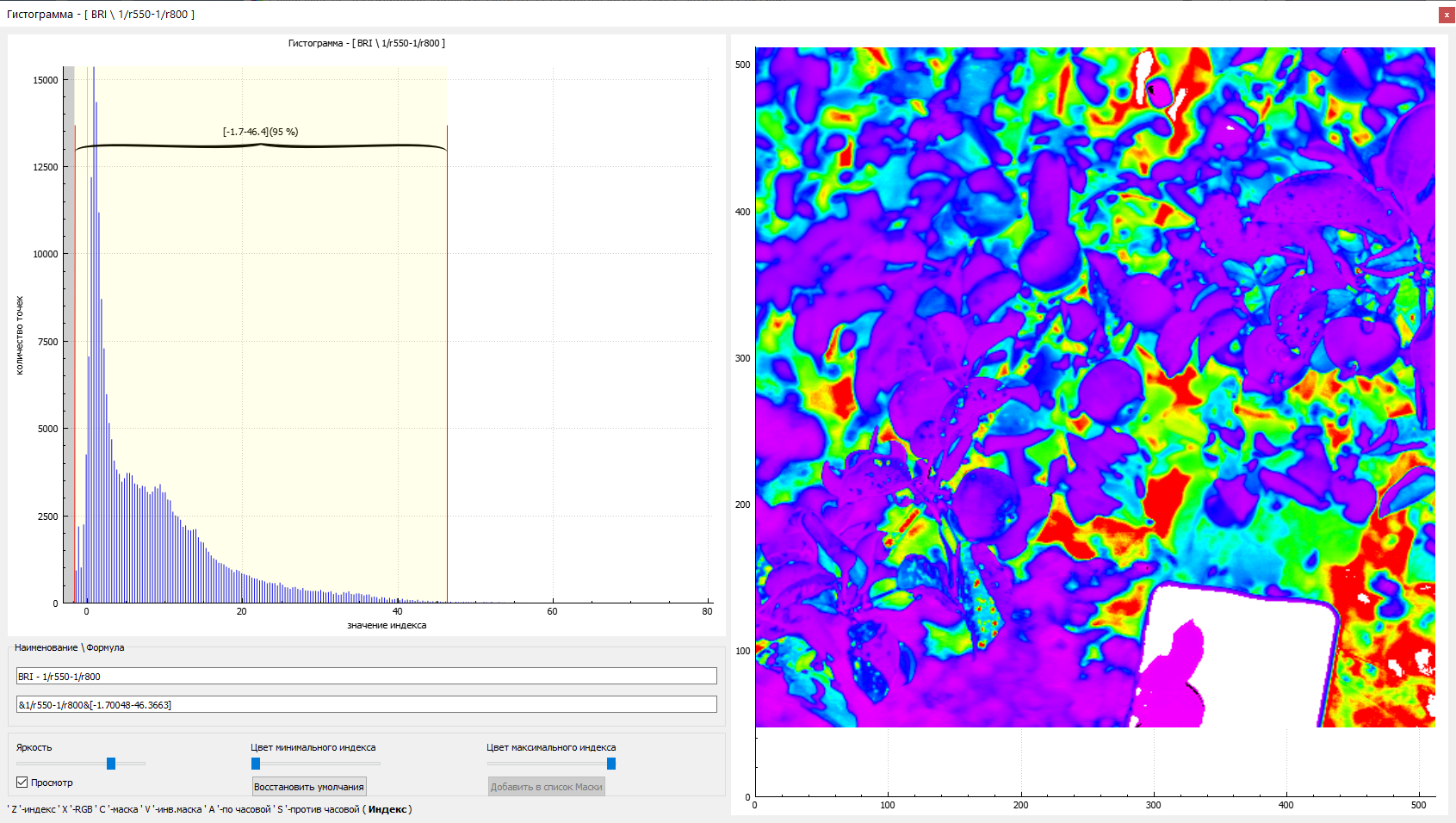  (**b**) |
| 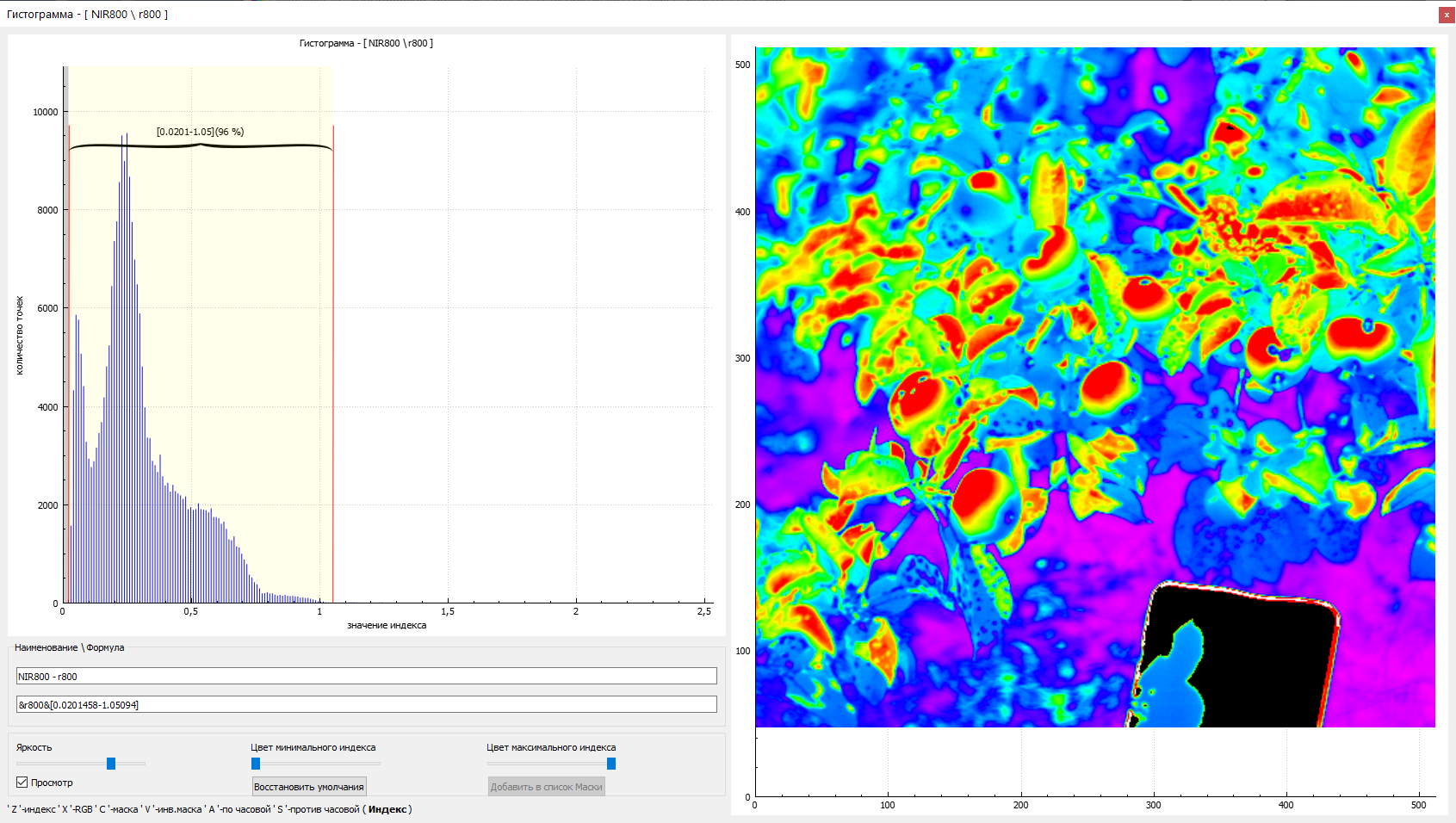  (**c**) |

**Figure S4.** Alternative representations of the scene shown on Fig. 6 in the main text in (**a**) a NIR reflectance channel, *R*_800_; (**b**) the index BRI [see Chivkunova et al. 2001], and (**c**) the index NDVI.
